# Supplementary material for: Utilizing a mixed-methods approach to assess implementation fidelity of a group antenatal care trial in Rwanda
Source: PLoS One. 2023 Jul 24;18(7):e0288974. doi: 10.1371/journal.pone.0288974 (PMC10365308; doi:10.1371/journal.pone.0288974)
Supplement: S1 Table — (DOCX) [file pone.0288974.s002.docx]

S1 Table. Multivariate Regression of Overall MFA Score and Individual MFA Items on Elapsed Time, Provider and Health Center Characteristics (N = 160)

| MFA Label |  | Average score | Room Setup | Facilitator Communication | Correct Assessments |
| --- | --- | --- | --- | --- | --- |
| MFA # |  |  | 4 | 5 | 6 |
| **Elapsed Time** | Coeff (SE) | .40 (.075) | 0.51 (.125) | .48 (.117 | .21 (.105 |
|  | Significance | *** | *** | *** | * |
| Provider Age | Coeff (SE) | -.01 (.008) | -.01 (.013) | -.01 (.012) | -.003 (.011) |
|  | Significance | .24 | .45 | .29 | .79 |
| Provider Education | Coeff (SE) | -.11 (.101 | -.19 (.172) | -.05 (.158) | -.12 (.143) |
|  | Significance | .29 | .27 | .73 | .41 |
| Provider Experience | Coeff (SE) | .004 (.009) | .01 (.014) | .01 (.013) | .01 (.012) |
|  | Significance | .65 | .52 | .35 | .51 |
| Urban (vs Rural) | Coeff (SE) | -.10 (.091 | -.12 (.153) | -.01 (.142) | -.30 (.128) |
|  | Significance | .27 | .42 | .92 | * |
| Patient to Staff Ratio | Coeff (SE) | -.005 (.006 | -.01 (.010) | -.01 (.009) | -.01 (.01) |
|  | Significance | .42 | .16 | .16 | .31 |

| MFA Label |  | Encouraged Participation | Promoted Discussion | Praised Group | Group Participation |
| --- | --- | --- | --- | --- | --- |
| MFA # |  | 7 | 8 | 10 | 11 |
| **Elapsed Time** | Coeff (SE) | .28 (.106) | .28 (.114) | .47 (.154) | .23 (.113) |
|  | Significance | ** | * | ** | * |
| Provider Age | Coeff (SE) | -.04 (.011) | -.02 (.012) | -.01 (.016) | -.02 (.011) |
|  | Significance | *** | * | .55 | .10 |
| Provider Education | Coeff (SE) | -.15 (.143) | .06 (.155) | -.22 (.209) | -.15 (.153) |
|  | Significance | .30 | .72 | .29 | .32 |
| Provider Experience | Coeff (SE) | .01 (.012) | -.02 (.013) | .003 (.018) | .004 (.013) |
|  | Significance | .31 | .20 | .88 | .77 |
| Urban (vs Rural) | Coeff (SE) | .22 (.129) | .30 (.139) | -.01 (.188) | -.05 (.134) |
|  | Significance | .09 | * | .95 | .70 |
| Patient to Staff Ratio | Coeff (SE) | -.005 (.008) | .02 (.009) | -.004 (.012) | .004 (.009) |
|  | Significance | .55 | * | .73 | .67 |
| MFA Label |  | Curriculum Knowledge | Responsive | Kept Time | Proper Screening |
| MFA # |  | 12 | 13 | 14 | 15 |
| **Elapsed Time** | Coeff (SE) | .59 (.126) | .48 (.109) | .51 (.190) | .36 (.115) |
|  | Significance | *** | *** | ** | ** |
| Provider Age | Coeff (SE) | -.01 (.013) | -.001 (.011) | .002 (.019) | .01 (.012) |
|  | Significance | .60 | .89 | .91 | .27 |
| Provider Education | Coeff (SE) | -.03 (.171) | -.27 (.148) | -.07 (.253) | -.24 (.155) |
|  | Significance | .85 | .07 | .77 | .12 |
| Provider Experience | Coeff (SE) | -.02 (.014) | -.01 (.012) | .03 (.021) | .003 (.013) |
|  | Significance | .21 | .30 | .21 | .81 |
| Urban (vs Rural) | Coeff (SE) | .09 (.152) | -.07 (.134) | -.32 (.23) | -.35 (.140) |
|  | Significance | .55 | .61 | .16 | * |
| Patient to Staff Ratio | Coeff (SE) | -.01 (.010) | -.001 (.009) | -.02 (.015) | -.005 (.009) |
|  | Significance | .30 | .95 | .25 | .91 |

Standard Errors in parentheses. *** p < 0.001; ** p < 0.01; * p < 0.05
