# Supplementary figures and images for: Utilizing a mixed-methods approach to assess implementation fidelity of a group antenatal care trial in Rwanda
Source: PLoS One. 2023 Jul 24;18(7):e0288974. doi: 10.1371/journal.pone.0288974 (PMC10365308; doi:10.1371/journal.pone.0288974)

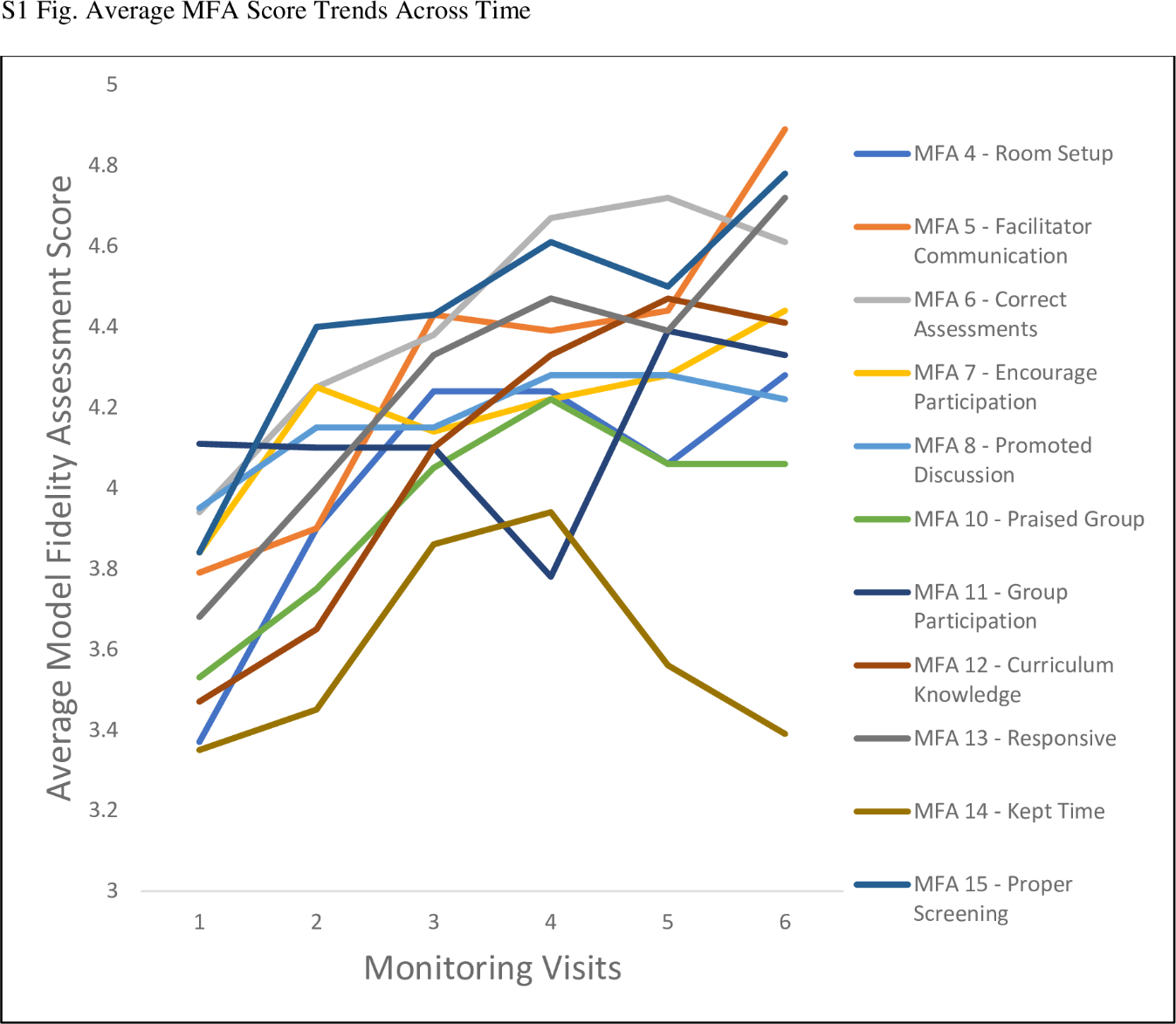

Supplement: S1 Fig — (DOCX) [file pone.0288974.s003.docx]
